# Filtenna with Frequency Reconfigurable Operation for Cognitive Radio and Wireless Applications

**DOI:** 10.3390/mi14010160

**Published:** 2023-01-08

**Authors:** Mahmoud A. Abdelghany, Wael A. E. Ali, Hesham A. Mohamed, Ahmed A. Ibrahim

**Affiliations:** 1 Electrical Engineering Department, College of Engineering, Prince Sattam Bin Abdulaziz University, Wadi Addwasir 11991, Saudi Arabia; 2 Electronics and Communications Engineering Department, Minia University, El-Minia 61519, Egypt; 3 Electronics and Communications Engineering Department, Arab Academy for Science, Technology & Maritime Transport (AASTMT), Alexandria 21937, Egypt; 4 Electronics Research Institute, Microstrip Circuits Joseph Tito St, Huckstep, El Nozha, Cairo 11843, Egypt

**Keywords:** filtenna, reconfigurable, varactor diode, IoT, cognitive radio

## Abstract

A reconfigurable wideband monopole antenna is introduced in this paper for cognitive radio and wireless applications. The reconfigurability was achieved by four varactor diodes embedded in the band pass filter (BPF) structure which was integrated with the suggested antenna through its feed line. The simulated impedance characteristics coped with the measured ones after fabricating the suggested model with/without the reconfigurable BPF. Furthermore, the model achieved the desired radiation characteristics in terms of radiation pattern with acceptable gain values at the selected frequencies within the achieved frequency range (1.3–3 GHz).

## 1. Introduction

All modern wireless systems require an antenna as a prominent part for transmitting and receiving electromagnetic waves efficiently which possesses wide impedance characteristics with improved radiation characteristics [1]. The Microstrip antenna is considered one of the favorable antennae utilized for various wireless communication systems due to its inherent advantages over other types of antennae such as compact dimensions, lightweight, less fabrication complexity and cost, and conformal structure on various objects [2,3]. Different wireless technologies are covered by the aforementioned wireless systems such as GSM1800 (1710–1880 MHz), GSM1900 (1850–1990 MHz), UMTS (1920–2170 MHz), LTE2300 (2305–2400 MHz), LTE2500 (2500–2690 MHz), and IEEE 802.11 b/g (2.4–2.48 GHz) [4,5]. To widen the operating bandwidth of microstrip antennae, many efforts have been exerted by the publishers taking into consideration the size miniaturization, complexity minimization, and cost reduction. Different techniques have been addressed to meet the desired requirements and some of these techniques are partial ground plane [6,7,8], electromagnetic band gap (EBG) ground structure [9], loaded rectangular and annular notches in the ground plane [10,11,12], radiator ring slot and shorting vias [13], parasitic elements [14], and electrostrictive effect [15].

The internet of things (IoT) is considered one of the most technological innovations that pave the way for human-machine interconnectivity which aims to communication devices within their environment through the internet with a high level of intelligence [16,17]. IoT operates in the frequency range from lower frequencies up to 5.8 GHz and microstrip antennae can be easily integrated into IoT devices, so the size miniaturization is considered an important factor in selecting the appropriate antenna model for IoT applications [18,19]. Another innovative technology that has been utilized to make the best use of the frequency spectrum is called cognitive radio (CR). In the CR system, the primary users are assigned to specific frequency bands while the secondary users can access the unoccupied frequency band which will allow spectrum sharing when it is not in use and this is controlled by a spectrum sensing process [20,21]. Consequently, a reconfigurable microstrip antenna with a wide frequency band is required for continuous monitoring of the unused spectrum in the CR system. Several studies have been conducted to reconfigure various frequency bands. A combination of dual-band and UWB antennae is utilized as communicating and sensing antennae for CR applications, respectively. The reconfiguration is accomplished using two PIN diodes connected at the communicating antenna [22]. Two PIN diodes are embedded in the UWB antenna to achieve band-notched behavior at the operating frequencies of WiMAX, WLAN, and ITU [23]. In [21], a CPW monopole antenna was used as a sensing antenna, and three narrow-band antennae covered seven sub-bands as communicating antennae, and one of them was a reconfigurable planar monopole antenna. A reconfigurable multiband monopole antenna was presented in [24] for triple/quadruple operations. A wide/dual/single band PIN-based reconfigurable monopole antenna with a band of operation (3.3–7.8 GHz) was introduced in [25]. A reconfigurable UWB monopole antenna was introduced in [26] for cognitive radio applications using six switches to obtain five narrow bands.

In this paper, a wide band antenna was integrated with reconfigurable BPF to achieve a reconfigurable filtenna for cognitive and wireless applications. The antenna was composed of a rectangular patch with a partial ground plane to cover the range from 1.3 to 3 GHz, and the BPF was integrated with four varactor diodes to pass a narrow band range within the aforementioned frequency range. The simulation outcomes are carried out on a computer simulation tool (CST) and the suggested model is fabricated to validate the simulation outcomes.

## 2. Second Order BPF Design

The first part of designing filtenna is the BPF, so a realization of the second-order BPF was investigated. Two configurations were presented to produce the desired band. The first one (filter 1) was the second-order BPF with a 180° feed layout, while the second one (filter 2) was the second-order BPF with 0° feed layout. The coupling between the two resonators and the external quality factor was extracted by utilizing the optimization technique. Therefore, the reduction of the objective function was considered the main part to obtain the needed coupling matrix between resonators. Equation (1) shows the desired objective function which needed to be minimized [27,28].

(1) $$\begin{array}{l} K={\sum_{i=1}^{N}|S_{11}(\omega_{zi}^{\prime })|}^{2}+{\sum_{i=1}^{P}|S_{21}\left(\omega_{pi}^{\prime }\right)|}^{2}+{\left(|S_{11}(\omega^{\prime }=-1)|-\frac{\varepsilon }{\sqrt{\begin{array}{cc} 1+ & \varepsilon^{2} \end{array}}}\right)}^{2}+\left(|S_{11}(\omega^{\prime }=1)|-\frac{\varepsilon }{\sqrt{\begin{array}{cc} 1+ & \varepsilon^{2} \end{array}}}\right) \end{array}$$

$$F_{N}\left(\omega^{\prime }\right)=\cosh\left(\sum_{n=1}^{N}\cosh^{-1}\left(\frac{\omega^{\prime }-1/\omega_{n}^{\prime }}{1-\omega_{}^{\prime }/\omega_{n}^{\prime }}\right)\right)$$

(2) ω′=ω0Δω(ωω0)−(ω0ω)

The poles and zeros of (*F_N_*) are *ω_pi_′, ω_zi_′*, where the *F_N_* can be obtained from Equation (2) [26]. At this stage, the *S_11_* and *S_21_* can be extracted using Equations (3) and (4) [27].

(3) $$S_{21}=-2j\sqrt{R_{1}R_{2}}{\left[A^{-1}\right]}_{N1},\;\left[A\right]=[\omega^{\prime }U-jR+M]$$

(4) $$S_{11}=1+2jR_{1}{\left[A^{-1}\right]}_{11}$$

where *R_1_*, R_2_, *M*, and *U* are I/O resistances, coupling matrix, and identity matrix, respectively.

To design the desired filter with 4.6 GHz central frequency, 400 MHz bandwidth, and −13 dB reflection coefficients, the previously investigated technique was utilized. The optimization technique with the quasi-Newton algorithm was used to minimize the objective function using Matlab (software 2015). The process needed nine iterations to extract the coupling matrix and external quality factor. The normalized coupling matrix which achieved the desired filter requirement was as in Equation (5).

(5) $$M=\left[\begin{array}{cc} 0 & 1.1691\\ 1.1691 & 0 \end{array}\right]$$

As well, the I/O external quality factors were 0.931. The actual coupling matrix is calculated as (6).

(6) $$m=M\times FBW=\left[\begin{array}{cc} 0 & 0.1017\\ 0.1017 & 0 \end{array}\right]$$

**Figure 1:**
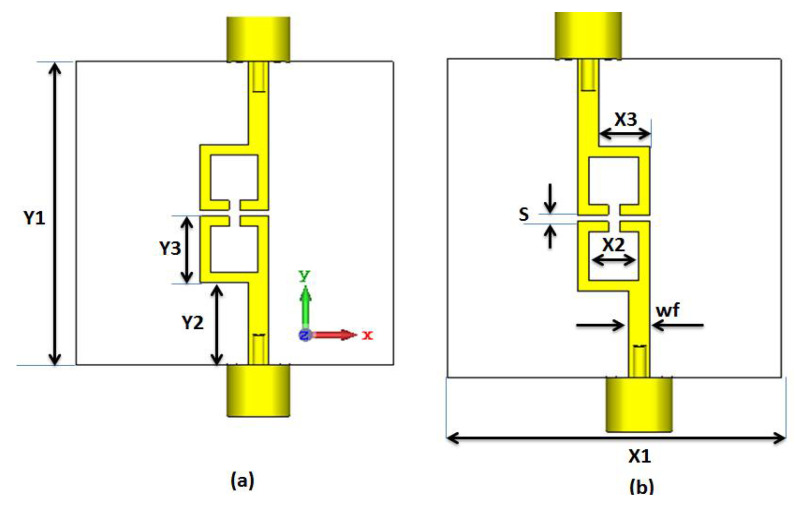
2-D layout of the second order BPF with Y1 = 30, Y2 = 8.2, Y3 = 6.5, X1 = 30, X2 = 4.5, X3 = 4.6, S = 0.6, and wf = 1.9 mm. (**a**) Filter 1 with 180° feed; (**b**) filter 2 with 0° feed.

The actual external quality factor was an external quality factor/FBW = 10.706. At this point, the BPF physical configuration from the filter syntheses can be realized based on the extracted coupling matrix and the external quality using CST Studio software. A Roger RO 4003 substrate with 3.38, and 0.813 mm dielectric constant, and thickness was utilized in the filter design. Figure 1a shows the second order with the 180° feed structure. The filter had two λ/2 capacitive coupling resonators. The coupling matrix can be validated by controlling the separation (S) between the resonators while moving the feed line around the X-axis was utilized to prove the external quality factor. The separation S was optimized to 0.6 mm and there was no distance between the feed line and the resonators in the simulation to achieve the desired coupling matrix and external quality factor.

**Figure 2:**
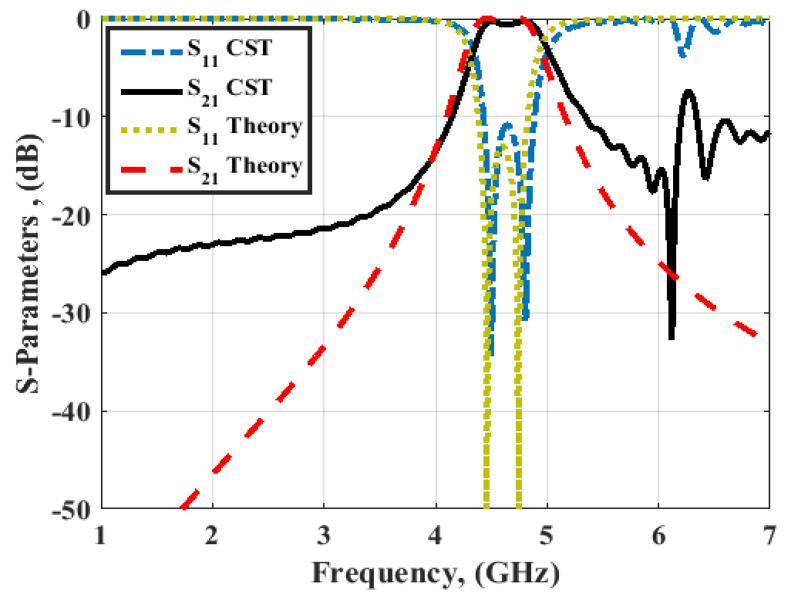
S-parameters outcomes of filter 1.

Figure 2 illustrates the S-parameters outcomes from the optimization technique (theory) and CST simulation of the BPF (Filter 1). The filter operated at 4.6 GHz central frequency, 0.41 GHz bandwidth, and −12 dB reflection coefficients. In addition, the simulated and the theoretical outcomes had the same trend.

**Figure 3:**
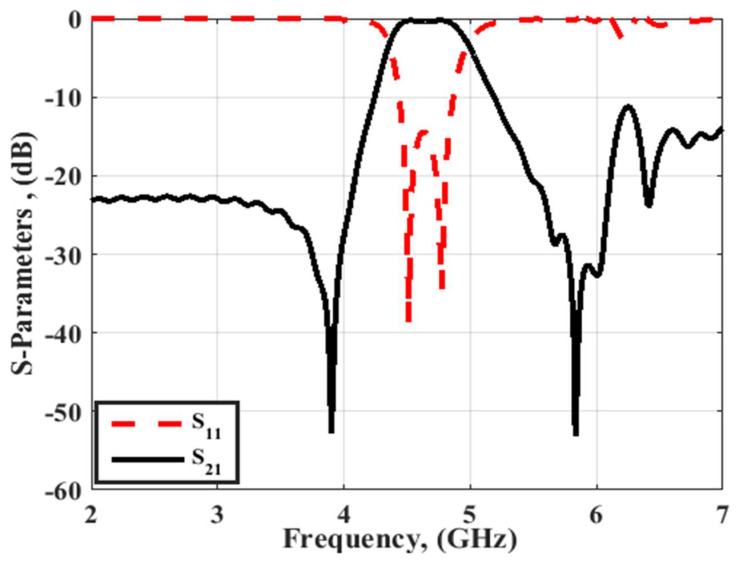
S-parameters outcomes of filter 2.

As illustrated in Figure 2, it is seen that the filter selectivity was low. Thus, to increase the filter selectivity, the number of resonators should be increased which in turn would increase the overall size of the filter, or the 0° feed structure can be employed as illustrated in Figure 1b. Figure 3 displays the S-parameters outcome of the 0° feed configuration (Filter 2). It is noticed that the filter had the same center frequency and bandwidth, while the two transmission zeros were obtained to enhance the filter selectivity at 3.9 and 5.9 GHz.

**Figure 4:**
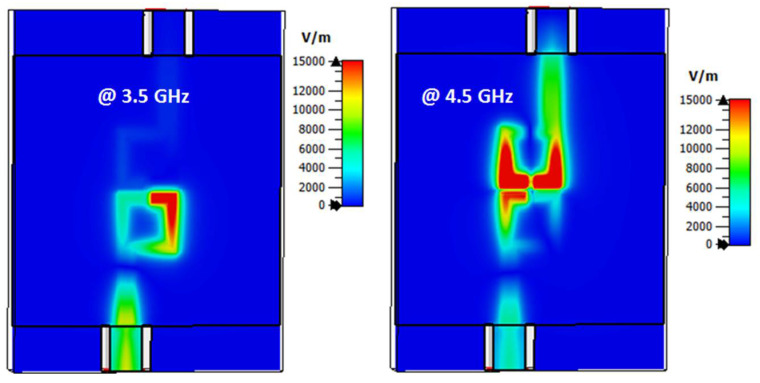
The electric field outcomes of filter 2 at different frequencies.

**Figure 5:**
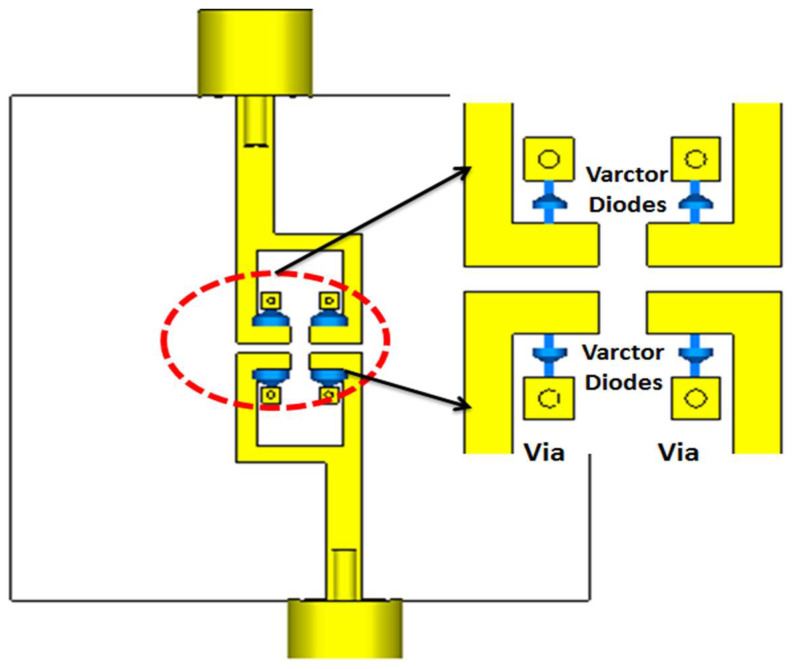
The 2-D layout of filter 2 with Varactor diodes.

**Figure 6:**
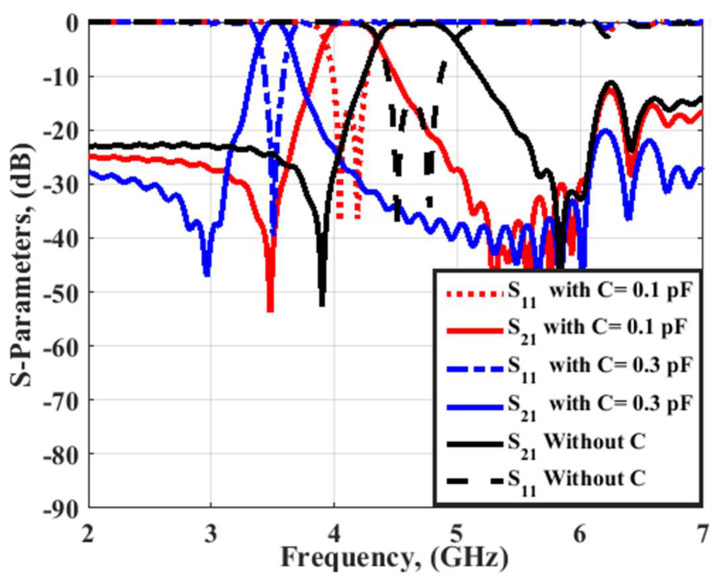
The S-parameters outcomes at different values of capacitance.

The electric field outcomes of filter 2 at 3.5 and 4.6 GHz are illustrated in Figure 4. As illustrated in Figure 3, the filter had a band stop operation at 3.5 GHz and passed the band operation at 4.5 GHz. Thus, the electric field was not transferred from port 1 to port 2 at 3.5 GHz; however, it was transferred at 4.5 GHz as illustrated in Figure 4. In addition, the electric field had a maximum level around the resonator’s gap at 4.5 GHz. Thus, by inserting four varactor diodes vertically near the gaps of the resonators as illustrated in Figure 5, the filter center frequency could be changed and controlled. Figure 6 shows the S-parameter outcomes at different values of the capacitance. The filter center frequency was moved from 4.6 GHz without capacitance to 4.1 GHz with 0.1 pF and to 3.5 GHz with 0.3 pF, respectively. The energy stored in the resonators was increased when inserting varactor diodes because they enhanced the resonator quality factor, and then the achieved bandwidth was reduced as shown in Figure 6. Therefore, the varactor diode’s capacitance could decrease the filter center frequency, but at the expense of its bandwidth and optimization should be done to extract the desired operation.

## 3. Antenna with Wide Band Operation

**Figure 7:**
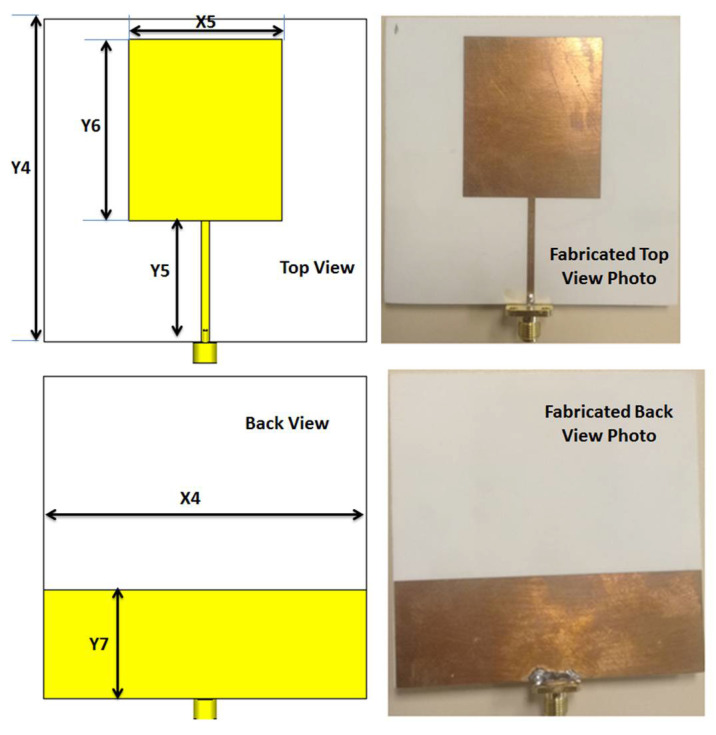
2-D layout of the wide band antenna with Y4 = 80, Y5 = 30, Y6 = 45, Y7 = 27, X4 = 80, X5 = 38 mm.

**Figure 8:**
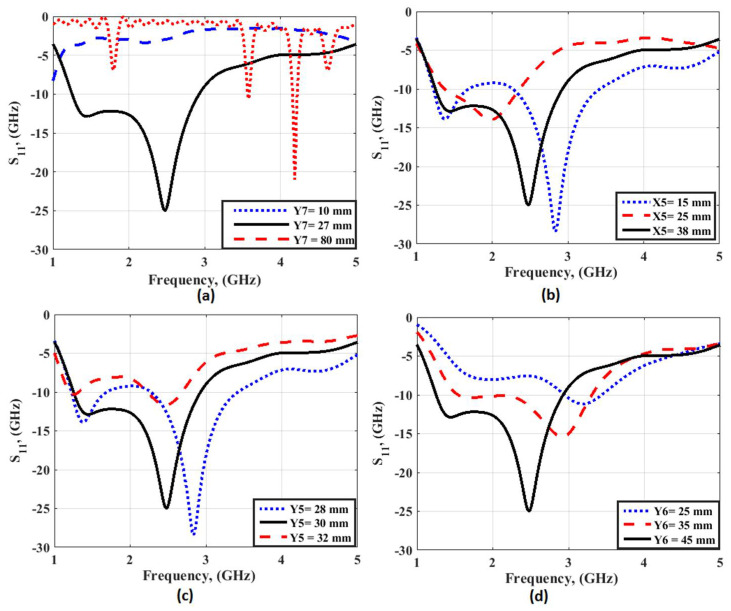
Parametric investigation on the antenna lengths: (**a**) Ground length (Y7); (**b**) patch width (X5); (**c**) feed line length (Y5); (**d**) patch length (Y6).

**Figure 9:**
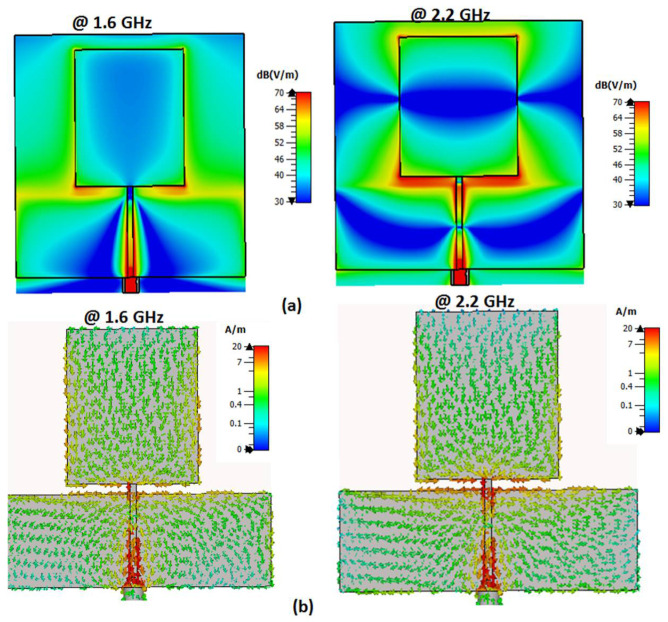
The antenna field distributions: (**a**) The electric field; (**b**) the surface current.

**Figure 10:**
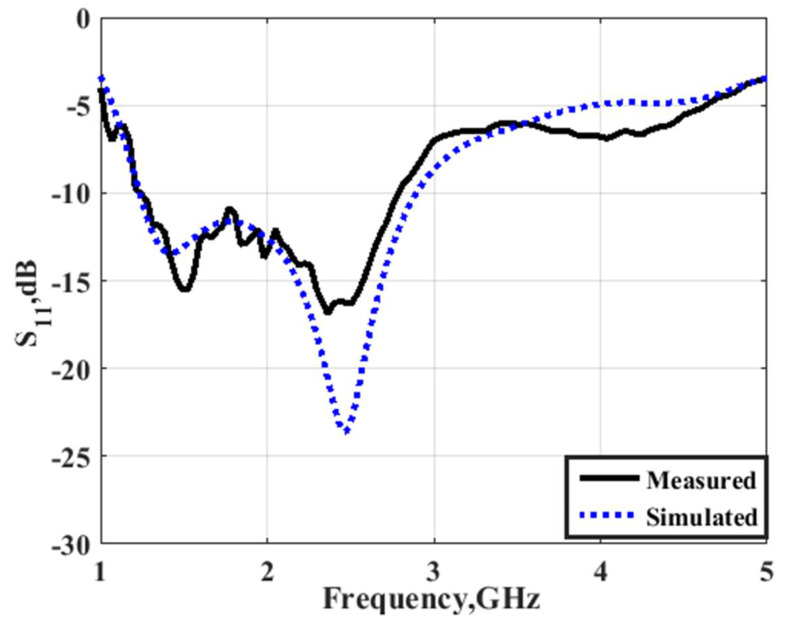
S_11_ simulated and measured outcomes.

The second part of the filtenna was the antenna, so a conventional monopole antenna with wide-band operation was utilized as illustrated in Figure 7. A rectangular patch with a partial ground plane was considered the main part of the monopole antenna. To enhance the antenna operation, the partial ground plane length should be optimized. To select the optimized dimensions of the monopole antenna shown in Figure 7, parametric investigation on the antenna lengths were carried out as illustrated in Figure 8. The parametric study was carried out when other lengths are fixed as illustrated in the caption of Figure 7. It is seen that the antenna achieved the proposed response when the antenna lengths equaled Y7 = 27, X5 = 38, Y5 = 30, and Y6 = 45 mm, respectively. The antenna electric field and the surface current distributions at 1.6 and 2.2 GHz are shown in Figure 9 which shows that the current was concentrated around the radiating patch. The antenna was operated with S_11_ ≤ −10 dB from 1.3 to 3 GHz, and this frequency band was chosen to be utilized in our CR system to sense and transmit the signals in the unoccupied channel easily. The previous substrate was utilized in the antenna design. The antenna was fabricated and tested using the Rohde & Schwarz (R&S ZVA 67) vector network analyzer (VNA) operated up to 67 GHz. Figure 10 displays the S_11_ simulated and tested outcomes. It was seen that the antenna had tested outcomes with S_11_ ≤ −10 dB from 1.3 to 3 GHz with a good trend with the simulated one.

## 4. The Suggested Filtenna

**Figure 11:**
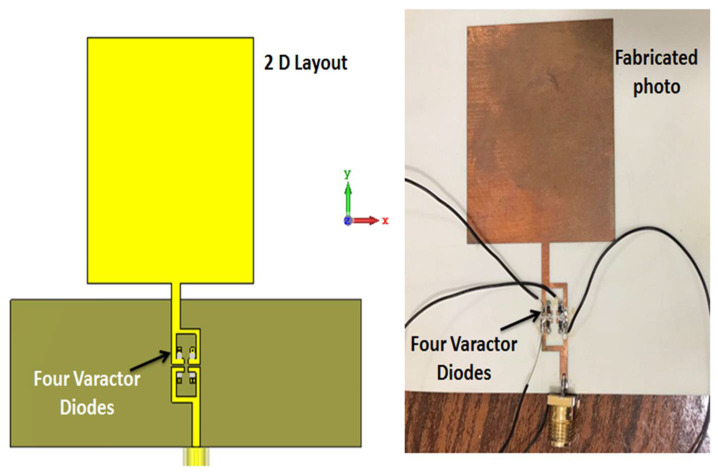
The configuration of the suggested filtenna.

At this stage, the BPF with four varactor diodes was embedded with the monopole antenna to compose the suggested filtenna. Figure 11 illustrates the 2-D layout and the fabricated photo of the filtenna. Four varactor diodes (MGV125-23) with a tuning range from 2.2 to 0.22 pF were utilized when external DC voltage was tuned from 0 to 20 V. The R&S ZVA 67 VNA was used in the testing.

**Figure 12:**
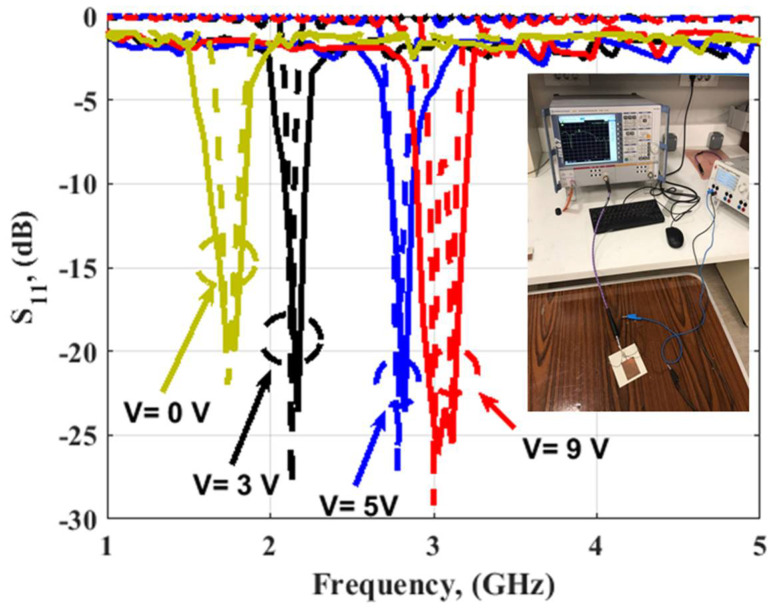
The S_11_ simulated (dashed) and measured (solid) outcomes of the filtenna at different V.

**Figure 13:**
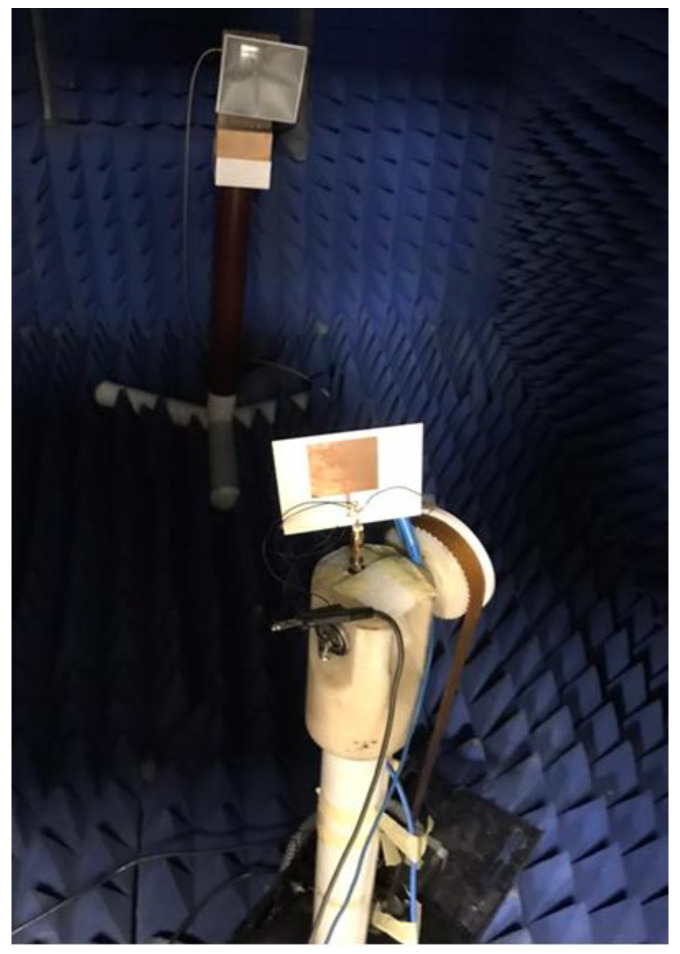
The setup of the radiation patterns measurements of the filtenna.

**Figure 14:**
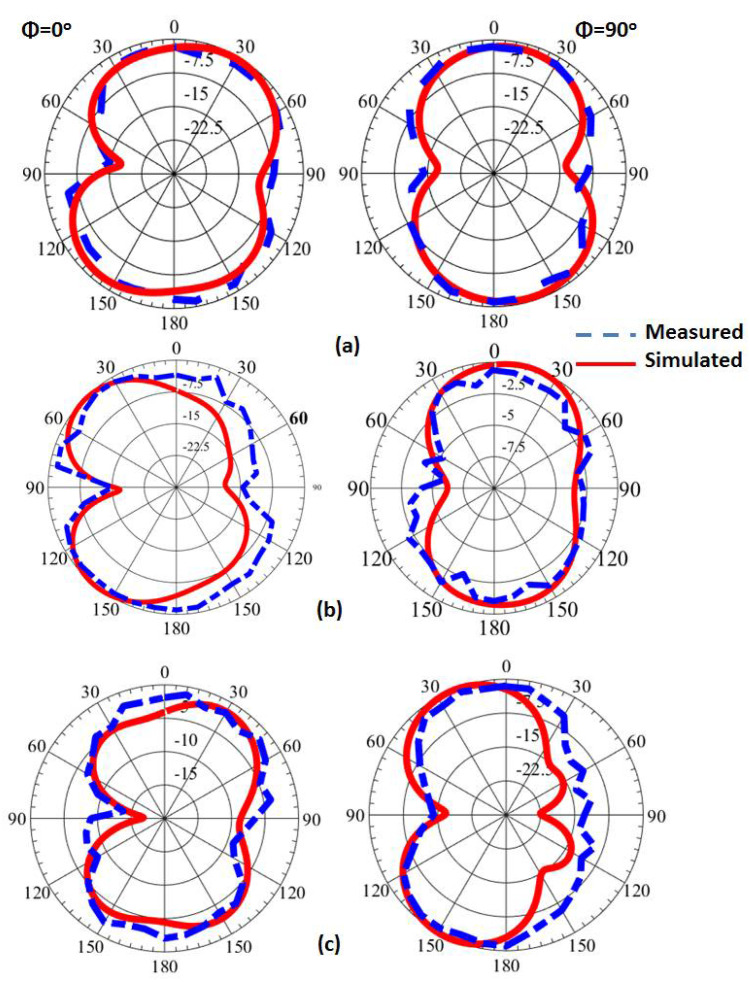
The normalized field patterns outcomes of the filtenna (**a**) @ 2.1 GHz and 3 V; (**b**) @ 2.8 GHz and 5 V; (**c**) @ 3 GHz and 9 V.

Figure 12 illustrates the filtenna simulated and measured outcomes at different voltages. When V = 9 volts, the capacitance equaled 0.5 pF, and the S_11_ was operated at 3 GHz with bandwidth with S_11_ ≤ −10 dB extended from 2.97 to 3.14 GHz for the simulated outcome, while the tested outcomes showed that the antenna was operated at 3 GHz with a bandwidth from 2.9 to 3.19 GHz. In addition, when V = 5 volts the varactor diode capacitance equaled 1 pF, and the S_11_ was operated at 2.78 GHz with bandwidth with S_11_ ≤ −10 dB extended from 2.74 to 2.83 GHz for the simulated outcome, while the tested outcomes showed that the antenna was operated at 2.8 GHz with a bandwidth from 2.74 to 2.78 GHz. When V = 3 volts the capacitance equaled 1.5 pF, and the S_11_ was operated at 2.13 GHz with a bandwidth with S_11_ ≤ −10 dB extended from 2.11 to 2.15 GHz for the simulated outcome, while the tested outcomes show that the antenna was operated at 2.16 GHz with a bandwidth from 2.1 to 2.2 GHz. Finally, when V = 0 volts the capacitance equaled 2.2 pF, and the S_11_ was operated at 1.74 GHz with a bandwidth with S_11_ ≤ −10 dB extended from 1.69 to 1.81 GHz for the simulated outcome, while the tested outcomes show that the antenna was operated at 1.75 GHz with a bandwidth from 1.64 to 1.83 GHz. Thus, the antenna operation was changed from wide band operation to narrowband operation at 3 GHz, 2.8, 2.1, and 1.74 GHz when the V was changed from 9 to 5, 3, and 0 V, respectively. In addition, the simulated and tested outcomes had good matching. The suggested filtenna was tested to extract the radiation patterns outcomes inside the anechoic chamber as shown in Figure 13. The normalized radiation patterns at 2.1 (3 V), 2.8 (5 V), and 3 GHz (9 V) were extracted in both φ = 0° and φ = 90 ° as illustrated in Figure 14. It is seen that the filtenna presented bidirectional patterns in both planes with a small shift between the two outcomes because of the measurement and fabrication process. The gain of the filtenna at the three frequency bands was extracted and achieved a simulated gain of 4.79 dBi and a tested gain of 4.65 dBi at 2.1 GHz. Additionally, it shows a simulated gain of 4.85 dBi and a tested gain of 4.54 dBi at 2.8 GHz. In addition, it had a simulated gain of 5.2 dBi and a tested gain of 5.05 dBi at 3 GHz. The filtenna was compared with the other design as tabulated in Table 1. It can be observed from the introduced antennae in Table 1 that the suggested single port filtenna used varactor diodes for the tuning range from 1.64 to 3 GHz, while other antennae with a different number of ports used PIN diodes and MEMS switched for a fixed number narrow band frequencies.

## 5. Conclusions

This work presented a reconfigurable filtenna for cognitive radio and wireless applications using four varactor diodes. The suggested antenna achieved a frequency range from 1.3 to 3 GHz then a reconfigurable BPF was integrated with the antenna to achieve the desired tunable frequencies of operation at 1.75, 2.1, 2.8, and 3 GHz when applying 0, 3, 5, and 9 voltages on the varactor diodes, respectively. Good consistency between the simulated and measured outcomes was observed after fabricating the proposed model and measuring it in an anechoic chamber. The suggested antenna succeeded to achieve the desired performance which makes it suitable for cognitive radio applications.

## Figures and Tables

**Table 1:** Comparison between the suggested model with recently reported antennae.

Ref.	Size (mm^2^)	Reconfiguration	Substrate	Number of Ports	Bands (GHz)
[21]	80 × 40	3 PIN diodes	FR4 (ε_r_ = 4.4)	4	3.863, 4.664, 5.2, 5.834, 6.13, 7.355, 8.786
[22]	50 × 70	2 PIN diodes	FR4 (ε_r_ = 4.4)	2	2, 2.2, 3.25, 3.8, 4.3, 5.6
[23]	20 × 20	2 PIN diodes	FR4 (ε_r_ = 4.4)	1	3.6, 5.5, 8.1
[24]	30 × 30	-	FR4 (ε_r_ = 4.4)	1	2.37, 4.1, 7, 9.76, 3.5, 7.2, 11.2
[25]	25 × 15	2 PIN diodes	FR4 (ε_r_ = 4.4)	1	3.5, 3.8, 6.1, 4–7.8
[26]	40 × 40	6 MEMS	FR4 (ε_r_ = 4.4)	1	5.8, 4, 5.6, 7.2, 7.8
proposed	80 × 80	4 Varactor diodes	RO4003 (ε_r_ = 3.38)	1	3, 2.8, 2.16,1.75

## Data Availability

All data generated or analyzed during this study are included in this article.
